# Clinical and serological association of plasma 25-hydroxyvitamin D (25(OH)D) levels in lupus and the short-term effects of oral vitamin D supplementation

**DOI:** 10.1186/s13075-022-02976-7

**Published:** 2023-01-03

**Authors:** Chengappa Kavadichanda, Pratibha Singh, Supriya Maurya, Sneha Tota, Aberaame Kiroubagarin, Deepika Kounassegarane, Swathi Anand, Vir Singh Negi, Amita Aggarwal

**Affiliations:** 1 Department of Clinical Immunology, Jawaharlal Institute of Postgraduate Medical Education and Research, Puducherry, India; 2 Department of Clinical Immunology and Rheumatology, Sanjay Gandhi Postgraduate Institute of Medical Sciences, Lucknow, India; 3 All India Institute of Medical Sciences, Bilaspur, Himachal Pradesh India

**Keywords:** Cholecalciferol, Autoimmunity, Interferon, SLE, SLEDAI-2KG

## Abstract

**Background and objectives:**

Data on the association of vitamin D levels and clinical phenotype and disease activity in systemic lupus erythematosus (SLE) is controversial. Further, the optimal dose of oral vitamin D supplementation in SLE is not clear. Thus, the present study was designed to determine the association of plasma vitamin D levels with clinical phenotype, disease variables and serology in a large, cohort of SLE from South Asia and to evaluate the short-term effect of two different dosage regimens of oral vitamin D supplementation on disease flares and plasma vitamin D levels.

**Methods:**

This is a two-phase study. Phase I was a cross-sectional analytical study of patients from north (26.85° N) and south India (11.94° N). Plasma 25-hydroxyvitamin-D(25(OH)D) was measured, and its association with demography, serology, disease activity, Galectin-9 and CXCL-10 was analysed. In phase II, patients with SLEDAI-2KG < 10 and on stable immunosuppression were randomised to receive either high dose (weekly 60,000 U*5, followed by 60,000 U monthly) or routine dose (30,000 U monthly) oral vitamin D. Outcomes were assessed at 6 months

**Results:**

Phase I included 702 patients with a mean age of 29.46 + 10.7 years. The median plasma vitamin D was 22.83 (13.8–31.8) ng/ml. Deficiency (< 20 ng/ml) was seen in 41.5% of patients. Patients from South India had higher vitamin D levels (27.06 ± 20.21 ng/dl) as compared to North India (17.15 ± 16.07 ng/ml) (*p* < 0.01). Univariate analyses demonstrated weak negative correlation of vitamin D with SLEDAI2K and positive correlation with age. Galactin-9 had modest correlation with SLEDAI2K but not with vitamin D levels. On multiple linear regression, centre of recruitment (*β* = 4.37) and age (*β* = 0.18) predicted (*p* < 0.05) plasma vitamin D levels.

In the phase II, 91 randomised to 2 groups completed 6 months. Median change in plasma vitamin D levels was more in high dose (9.5 versus 2.6 ng/ml; *p* = 0.04). There were 14 SLE flares and six minor adverse events which were equal across both groups.

**Conclusion:**

Vitamin D deficiency is common in SLE. Geographical location of residence is the major determinant rather than the disease activity. The IFN regulated proteins reflect disease activity independent of vitamin D levels. High-dose oral vitamin D supplementation seems safe and more effective in improving vitamin D levels in SLE.

**Trial registration:**

The second phase of this study was a registered randomised controlled trial CTRI/2019/06/019658 [registered on: 14/06/2019].

**Supplementary Information:**

The online version contains supplementary material available at 10.1186/s13075-022-02976-7.

## Introduction

Vitamin D and its link with autoimmune inflammatory diseases is an important ongoing area of research. The two critical unaddressed questions at present are does vitamin D level determine clinical phenotype in established autoimmune diseases like systemic lupus erythematosus (SLE)? Are the current oral vitamin D supplementation protocols in individuals with SLE successful and safe in improving plasma 25-hydroxyvitamin D (25(OH)D) levels along with additional clinical benefits?

In SLE, it is unclear if the low vitamin D levels cause autoimmunity or are the result of photoprotection [1], drugs used in the treatment of lupus or due to their role as a negative acute phase reactant [2]. Besides calcium homeostasis and bone health, 25-hydroxyvitamin D (25(OH)D) is important in maintaining immune homeostasis. Vitamin D enhances chemotaxis and phagocytosis of macrophages. It reduces IL-17 release by T cells, inhibits proliferation and differentiation of B cells and decreases the production of immunoglobulins [3, 4]. Lupus, on the other hand, has macrophage dysfunction, increased IL-17 production and autoantibody production. Thus, it seems that serum 25(OH)D levels have mechanistic links to the pathogenesis of SLE [5]. Several cross-sectional studies evaluating the association of vitamin D levels with lupus phenotype, disease activity and outcome have arrived at contradicting conclusions [6–10].

Hence, there is a need to evaluate the associations of vitamin D levels with disease activity and clinical phenotype across varying ethnic and geographical backgrounds in a large population. The prevalence of vitamin D deficiency is high among Asians [11]. India has a unique advantage of genetic diversity, which is reasonably ascertained by their native language. The country also boasts of contrasting geographical and environmental factors, a combined view of which enables us to determine the genetic background of individuals [12]. We intend to use this diversity to establish the association of vitamin D levels with clinical phenotype in lupus.

Recent studies have shown that orally supplementing vitamin D decreases progression to autoimmunity [13]. It is unclear if such supplementation will impact disease control in SLE. Moreover, the challenges of supplementing vitamin D in SLE are that the optimal safe dose that can replenish serum vitamin D levels is also not known [14]. So, before attempting to use vitamin D as an adjunct to immunosuppressive agents in SLE for disease control, it is necessary to establish the most effective route and dose of administration.

Hence, this study was designed with the following objectives. First is to study the relationship of vitamin D levels with disease variables and interferon-regulated proteins in patients with systemic lupus erythematosus (SLE) across two ethnic groups of patients from 2 different geographical locations in India. Second is to assess the effect of different dosage regimens of oral vitamin D supplementation on plasma vitamin D levels, disease status and flares at 6 months.

## Materials and methods

### Patients and study design

We conducted the study in two phases. Phase I was a cross-sectional study, and phase II was an interventional study. Patients with systemic lupus erythematosus who satisfied the SLICC 2012 classification criteria [15] were enrolled in the study. The patients were recruited at two centres, i.e. Lucknow (26.8467° N, 80.9462° E) and Pondicherry (11.9416° N, 79.8083° E) in India, respectively. The two areas differ in the amount of sunlight and ethnicity of people. The patients were either part of the Indian SLE inception cohort for research (INSPIRE) [16] (*n* = 530) or part of an oral vitamin D supplementation trial [clinical trial registry number: CTRI/2019/06/019658 [registered on: 14/06/2019] which had to be terminated prematurely due to COVID 19 pandemic (*n* = 172).

The respective institutional ethics committees approved the study, and we conducted the study in accordance with the declaration of Helsinki's guidelines for human research. All patients gave written informed consent.

Demographic, anthropometric and socioeconomic (SES) details per modified Kuppuswamy’s classification [17] of the patients were collected. Kuppuswamy’s classification of the socioeconomic status is a composite score which includes the education and occupation of the family head along with the income per month of the family. The parameters yield a score of 3–29 classifying the population into five SES, namely lower, upper-lower, lower-middle, upper-middle and upper socio-economic class.

Disease activity was assessed using SLE Disease Activity Index-2000 (SLEDAI-2K) [18], and damage was assessed by the Systemic Lupus International Collaborating Clinics/American College of Rheumatology (SLICC/ACR) Damage Index (SDI) [19]. All the patients who were part of the INSPIRE cohort had their blood samples collected on the day of enrolment into the cohort. The sampling was done between October 2018 and February 2022.

**Fig. 1:**
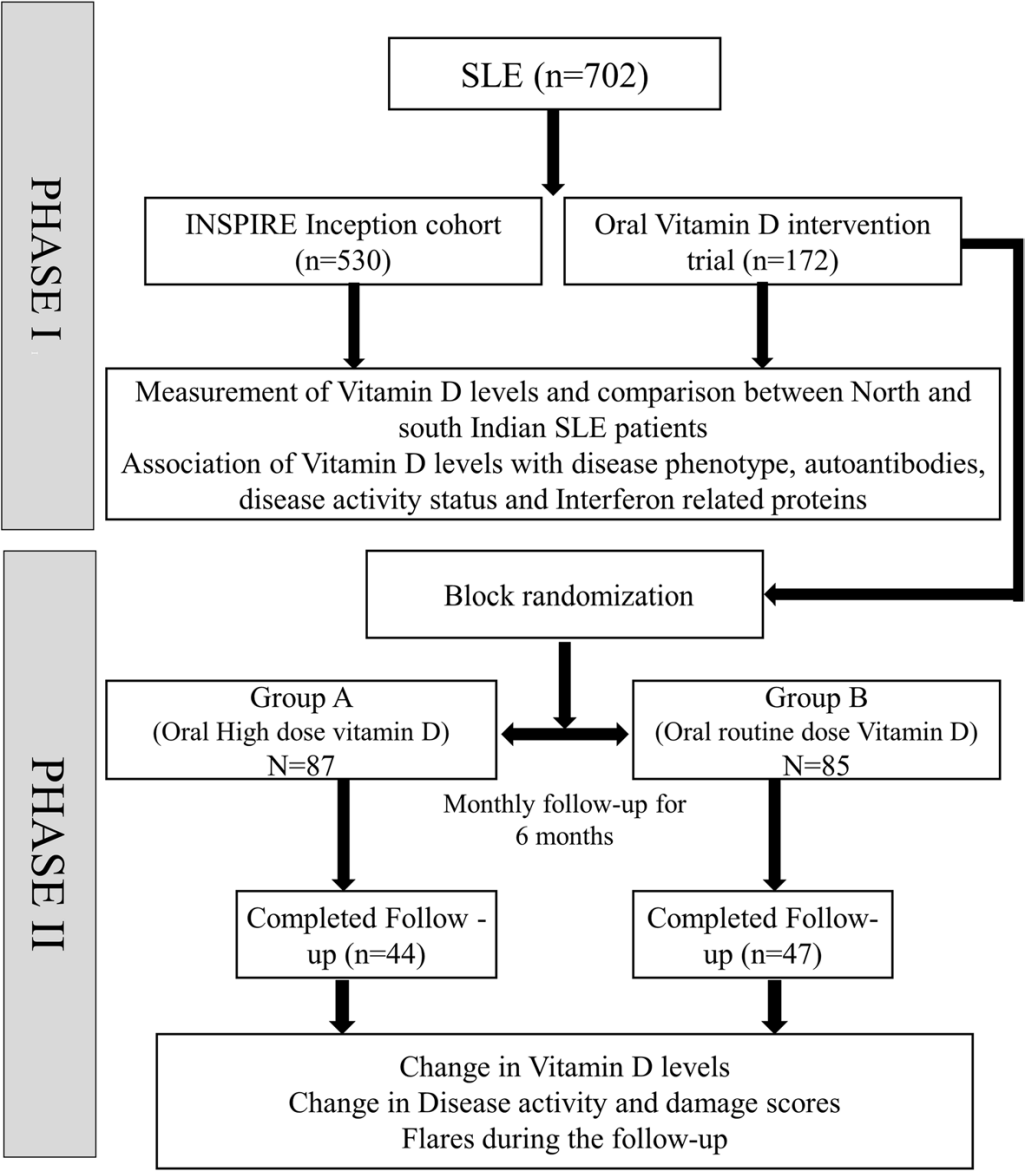
Flow diagram elaborating the details of the design and execution of the study

For the interventional study (phase II), SLE patients satisfying the SLICC 2012 classification criteria [15] above the age of 18 years, with SLEDAI 2KG < 10 [20] and on a stable dose of immunosuppressive drugs for 3 months prior to inclusion. All the patients who were part of the trial (phase II) were on calcium and vitamin D supplements and were recruited between January 2019 and February 2020. The patients were then randomised using permuted block randomisation method into two groups (high dose and routine dose). The high dose group was given 5 weekly oral doses of vitamin D 60,000 units followed by monthly 60,000 units for 6 months, and the routine dose group received 30,000 units monthly for the same 6 months without the weekly dose. Patients were followed up every 3 months to document disease flare as defined by the SLEDAI flare index [21]. Vitamin D levels, anti-dsDNA antibody, SLEDAI-2K and the number of flares of the patients were evaluated monthly, and the changes were compared between the two groups. The trial was terminated prematurely during the COVID pandemic (April 2020). The pictorial representation of the workflow for this study is depicted in Fig. 1.

### Laboratory assessment

Anti-nuclear antibody (ANA) was tested by indirect immunofluorescence (Hep 2010, EUROImmune, Lubeck, Germany). Serum complements C3 and C4 were measured using BN™ II System nephelometer (SIEMENS Healthliners). Anti-double stranded DNA (anti-dsDNA) was measured using ELISA (EUROImmune, Lubeck, Germany). In a subset of patients, interferon-related proteins galectin 9 and CXCL 10 were measured using ELISA (R&D system, USA). Plasma vitamin D levels were measured using chemiluminescence (COBAS, Roche Diagnostics GmbH, Mannheim, Germany). Vitamin D levels of ≤ 20 ng/ ml was defined as deficiency, > 20 to < 30 ng/ml as insufficiency and ≥ 30 ng/ml as sufficient [21].

### Statistics

Normality of data was ascertained by Kolmogorov Smirnov test. All continuous variables in this study were expressed as mean standard deviation (SD) or median interquartile range (IQR)/range as per the normality of the data. Categorical data was represented as number and percentage. Correlation among SLE disease activity scores, damage scores, levels of 25-hydroxyvitamin D3, CXCL-10, galectin-9 anti-dsDNA, serum complement components, age and disease duration was studied by Spearman’s rank correlation analysis. Association of categorical variables between two centres and between those with and without vitamin D insufficiency was tested using chi-square test or Fisher’s exact test. The continuous factors were tested by independent Student *T* test/Mann-Whitney *U* test depending upon the distribution of the variable. Using Spearman’s correlation, a multiple linear regression model was built to assess the predictors of vitamin D levels. The model included all the factors showing significant correlation (*p* < 0.05) with plasma vitamin D levels along with the categorical variable—study centre. Collinearity statistics was conducted. All the statistical tests were performed using SPSS V 19. Bivariate correlation was done using STATA

## Results

### Demography

For the phase I of the study, 702 patients with SLE were included of whom 663 (93%) were females. The mean age of the participants was 29.44 ± 10.7 years, and the median duration of disease was 16 [8–36] months (Supplementary Table 1). The median vitamin D levels in the plasma was 22.83 (13.8–31.8) ng/ml with 41.5% being vitamin D deficient and 29% having sufficient vitamin D levels. One patient had vitamin D level of 109.5 ng/ml, and none had vitamin D toxicity. The median SLEDAI was 10 [4–16] and 69% had SLEDAI-2K of more than 4.

### Effect of geographical location on vitamin D levels

To see the impact of difference in latitude on vitamin D levels, patients from the two centres were compared. Patients from north India had a longer median duration of illness 20 [37] versus 15 [23] months (*p* = 0.03) and higher proportion of juvenile patients (19.1%) versus the 11.3% in the south Indian centre. The median (IQR) vitamin D levels were 17.15 (16.07) ng/ml and 27.06 (20.21) ng/ml, *p* < 0.001, at the north and south Indian centres respectively. Vitamin D deficiency was seen in 50.33% of patients from the north Indian centre versus 34.76% in the south. Upon analysing juvenile and adult patients separately, vitamin D levels were similar among the juvenile patients from both centres (*p* = 0.53). Still, they were higher in the south Indian adult patients [25.5 (13.92) ng/ml] when compared to their north Indian counterparts (Table 1).

**Table 1:** Comparison of baseline clinical and serological variables along with plasma vitamin D levels between the north Indian and south Indian centres

Parameter	Overall Cohort		Juvenile SLE		Adult SLE	
	North India *n* = 304	South India *n* = 398	North India (*n* = 58)	South India (*n* = 45)	North India (*n* = 246)	South India (*n* = 353)
Age in years (mean ± SD)	28.75 ± 11.04	30.01 ± 10.45	15 ± 3.02	15.53 ± 2.6	30.26 ± 9.12	31.17 ± 10
Females (%)	283 (93.1%)	370 (92.9%)	53 (91.38%)	39 (86.67%)	230 (93.5%)	331 (93.77%)
Duration of SLE in months Median (IQR)	20 (39)	15 (23)*	11.5 (19)	12 (15)	24 (44.2)	15 25)*
BMI (mean ± SD)	21.36 ± 4.8	21.59 ± 5.1	16.7 ± 3.13	18.1 ± 3.99	21.64 ± 4.52	20.9 ± 4.6
Plasma vitamin D, median (IQR) ng/ml	17.15 (16.07)	27.06 (20.21)*	17.64 (14.31)	21.04 (19.31)	20.27 (17.3)	25.5 (13.92)*
Vitamin D deficient (≤ 20 ng/ml) (%)	153 (50.33%)	138 (34.76%)*	32 (55.17%)	20 (44.44%)*	121 (49.19%)	118 (33.43%)*
Vitamin D insufficient (20.1–29.9 ng/ml) (%)	87 (28.62%)	121 (30.33%)*	22 (37.93%)	13 (28.89%)	65 (26.42%)	108 (30.59%)
Vitamin D sufficient (≥ 30 ng/ml) (%)	64 (21.05%)	139 (35.01%)*	4 (6.9%)	12 (26.67%)	60 (24.39%)	127 (35.98%)
SLEDAI 2K at baseline, median (IQR)	12 (8)	13 (11)	15 (10)	14 (12)	11 (8)	13.5 (11)
SLEDAI 2K > 4 (*n*, %)	204 (67.11%)	280 (70.53%)	46 (79.31%)	41 (91.1%)	158 (64.23%)	239 (67.71%)
C3 mg/dl Median (IQR)	57.45 (51.45)	56.7 (53.5)*	48.5 (38.2)	73.5 (67.9)	65.5 (51.61)	62.7 (51.83)*
Low C3, *n* (%)	190 (62.5%)	257 (64.73%)	36 (62.07%)	33 (73.33%)	154 (62.6%)	224 (63.46%)
C4 mg/dl Median (IQR)	11.34 (12.49)	9.53 (9.17)	9.13 (12.17)	10.35 (22.49)	11.4 (12.9)	9.38 (8.75)
Low C4, *n* (%)	149 (49.01%)	168 (42.1%)	28 (48.28%)	23 (51.11%)	121 (41.19%)	145 (41.08%)*
Low complements (C3/C4), *n* (%)	212 (69.74%)	278 (70.02%)	41 (70.69%)	36 (80%)	171 (69.51%)	242 (68.55%)
Anti-dsDNA (IU/ml) Median (IQR)	158.11 (217.68)	521.67 (659.32)*	259 (235)	624 (641)*	136.4 (248)	458.6 (659)*
Positive anti-dsDNA, *n* (%)	210 (69.07%)	258 (64.74%)	41 (70.69)	35 (77.78)	169 (68.7)	223 (63.17)
CXCL-10 pg/ml, median (IQR) (*n* = 474)	223.96 (438.86)	138.69 (202.73)	293.26 (554.03)	178.69 (249.02)	207.13 (416.49)	130.28 (178.31)
Galectin 9 ng/ml, median (IQR) (*n*—548)	10.55 (11.45)	13.66 (17.20)*	9.99 (15.65)	11.79 (15.09)	10.78 (10.21)	14.03 (17.76)*
Renal, *n* (%)	141 (46.38%)	142 (35.76%)*	26 (44.83%)	19 (42.22%)	115 (46.75%)	123 (34.84%)*
CNS, *n* (%)	48 (15.79%)	65 (16.37%)	13 (22.41%)	5 (11.11%)	35 (14.23%)	60 (17%)
Mucocutaneous, *n* (%)	268 (88.15%)	366 (92.19%)*	53 (91.38%)	42 (95.56%)	215 (87.4%)	324 (91.78%)*

*BMI* body mass index, chemokine (C-X-C motif) ligand, *CNS* central nervous system, *dsDNA* double-stranded deoxyribose nucleic acid, *SLEDAI* systemic lupus erythematosus disease activity index, *p < 0.05

### Associations of vitamin D levels with clinical and serological factors

Patients with sufficient vitamin D levels had no difference in clinical manifestations, disease activity and levels of interferon-related proteins (CXCL-10 and Galectin 9) as compared to those with insufficient and deficient levels. The only difference was in age which was higher (30.81 ± 10.4 years) in the vitamin D sufficient group as compared to those with low vitamin D levels (27.22 ± 10.6 years; *p* < 0.05) (Table 2).

**Table 2:** Comparison of baseline variables between those with and without sufficient vitamin D levels

Parameter		Vitamin D < 30 mg/ml (***n*** = 499)	Vitamin D ≥ 30 mg/ml (***n*** = 203)	***P*** value
Age in years (Mean ± SD)		27.22 ± 10.6	30.81 ± 10.4	< 0.001*
Females (%)		465 (93.19%)	188 (92.61%)	0.449
Geographical location	North	240	64	< 0.001*
	South	259	139	
Socioeconomic status (*n* = 609)	Lower class	61/437	34/172	0.066
	Upper Lower	141/437	66/172	
	Lower middle	140/437	44/172	
	Upper middle	88/437	28/172	
	Upper class	7/437	0/172	
Duration of SLE in months Median (IQR)		12 (19)	11.5 (19)	0.682
BMI (mean ± SD)		20.27 ± 4.53	21.1 ± 4.77	0.124
SLEDAI 2K at baseline, median (IQR)		12 (11)	12.5 (10)	0.373
SLEDAI 2K > 4 (*n*, %)		352 (70.54%)	132 (65.02%)	0.113
C3 mg/dl Median (IQR)		56 (49.7)	61.8 (65.1)	0.180
Low C3, *n* (%)		321 (64.33%)	126 (62.07%)	0.551
C4 mg/dl Median (IQR)		10 (9.65)	10.4 (9.79)	0.260
Low C4, *n* (%)		230 (46.1%)	87 (42.86%)	0.44
Low complements (C3/ C4)		353 (70.74%)	137 (67.49%)	0.395
Anti-dsDNA (IU/ml) median (IQR)		300 (670)	314.15 (719)	0.326
Positive anti-dsDNA, *n* (%)		336 (67.33%)	132 (65.02%)	0.589
CXCL-10 pg/ml, median (IQR) (*n* = 474)		162.51 (263.22)	145.1 (242.57)	0.146
Galectin 9 ng/ml, median (IQR) (*n*—548)		11.80 (11.66)	14.97 (25.13)	0.525
Renal, *n* (%)		209 (41.88%)	74 (36.45%)	0.177
CNS, *n* (%)		85 (17.03%)	28 (13.79%)	0.289
Mucocutaneous, *n* (%)		445 (89.18%)	190 (93.60%)	0.071

*BMI* body mass index, chemokine (C-X-C motif) ligand, *CNS* central nervous system, *dsDNA* double-stranded deoxyribose nucleic acid, *SLEDAI* systemic lupus erythematosus disease activity index, *p < 0.05

**Fig. 2:**
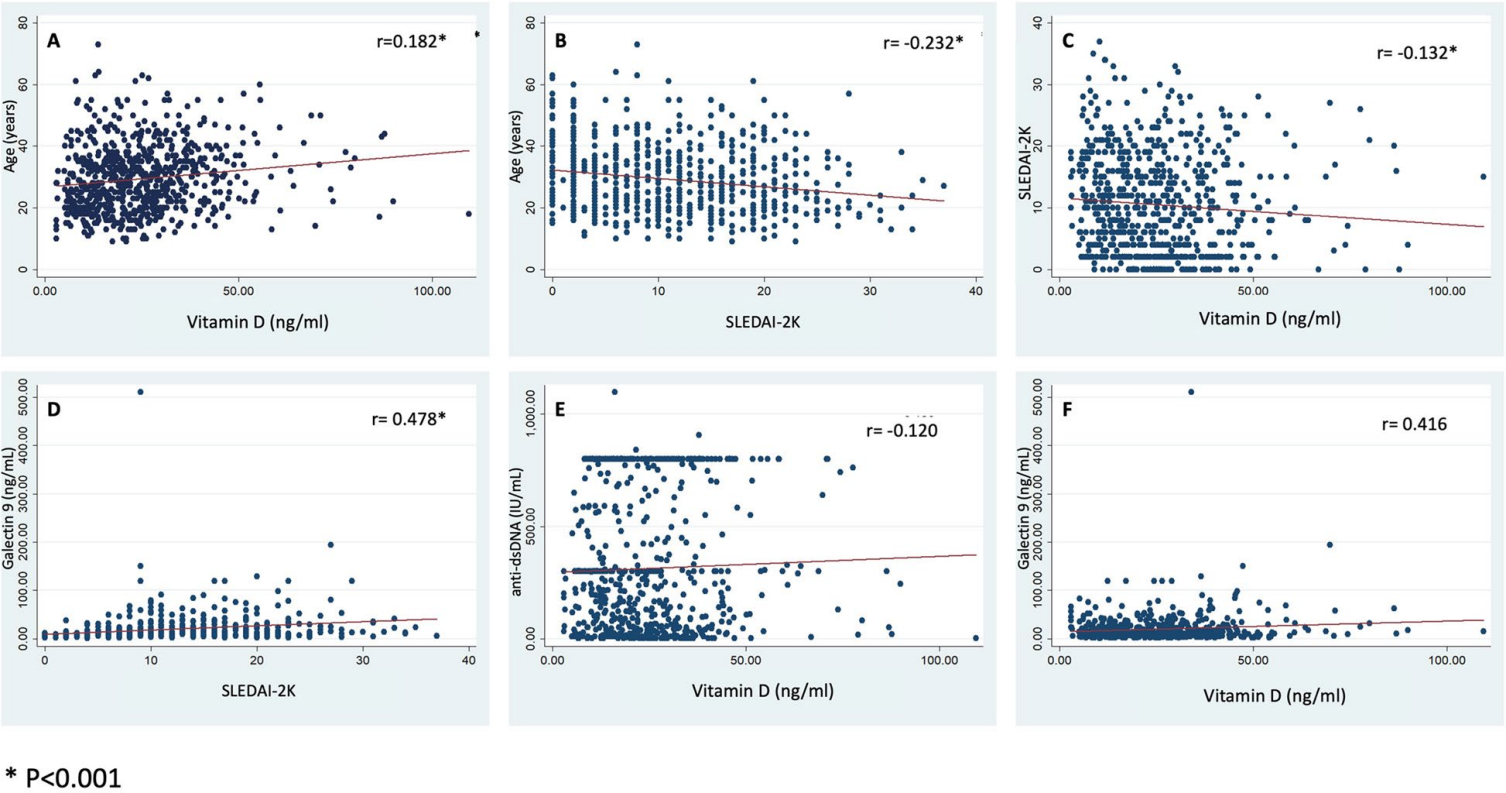
Spearman’s correlation between **A** age and plasma vitamin D levels, **B** age and SLEDAI-2K, **C** plasma vitamin D levels and SLEDAI-2K, **D** Galectin-9 and SLEDAI-2K, **E** anti dsDNA and plasma vitamin D and **F** Galectin-9 and SLEDAI-2K

Plasma vitamin D levels did not correlate with duration of disease, levels of anti-dsDNA, CXCL-10 and Galactin-9 (*p* > 0.05). However, it showed weak negative correlation with SLEDAI-2K (Spearman’s rho − 0.134, *p* < 0.001) and positive correlation with age (rho = 0.182, *p* < 0.001). SLEDAI-2K had weak negative correlation with age of the patients (rho = − 0.232, *p* < 0.001) and modest negative correlation with duration of illness (rho = − 0.453, *p* < 0.001). Galactin-9 had modest positive correlation with SLEDAI-2K (rho = 0.477, *p* < 0.001) (Fig. 2).

A multiple linear regression model was built taking all the variables that had significant correlation (*p* < 0.05) with vitamin D. The centre of treatment, which reflected the patients’ geographical location and ethnicity, was added to the model as an independent variable. When adjusted for all the variables, the centre of treatment had a *β* coefficient of 4.369 (*p* < 0.05) for predicting the plasma vitamin D levels (Supplementary Table 2).

In the phase II, a total of 172 patients were enrolled, with 87 in the high dose group and 85 in the routine dose group. The baseline features of all the patients in both the groups were similar (Supplementary Table 3). Due to the disruption in healthcare service during the COVID-19 pandemic, 6-month follow-up was available only for 91 individuals (44 in high dose and 47 in routine dose).

At 6 months, the median improvement in the plasma vitamin D levels was higher in high dose group 9.5 (14.5) ng/ml versus 2.6 (14.6) ng/ml in the routine dose group (*p* = 0.04). In the high dose group, 28 (63.6%) were vitamin D sufficient as opposed to 22 (46.8%) in the routine dose group at the end of 6 months (*p* = 0.02). There were 14 flares during the 6 months of follow-up with 5 major and 9 minor flares. The incidence of flare was similar across both the groups (Table 3). There were 6 non-serious adverse events in 5 patients in the study, including headache *n* = 1, vomiting *n* = 4 and diarrhoea *n* = 1 (3 in each group). There was no case of vitamin D toxicity.

**Table 3:** Outcome of 6 months of oral vitamin D supplementation

Parameter	High dose (***n*** = 44)	Routine dose (***n*** = 47)	***p*** value
Vitamin D at 6 months Median (IQR) ng/ml	34.12 (15.4)	34.61 (12.7)	0.16
Change in vitamin D over 6 months Median (IQR) ng/ml	9.5 (14.5)	2.6 (14.6)	0.04
Vitamin D deficient at baseline (< 20 ng/ml)	13	13	0.88
Vitamin D insufficient at baseline (20.1–29.9 ng/ml)	20	20	
Vitamin D sufficient at baseline (≥ 30 ng/ml)	11	14	
Vitamin D deficient at 6months (< 20 ng/ml)	6	2	0.021
Vitamin D Insufficient at 6months (21–29 ng/ml)	10	23	
Vitamin D sufficient at 6months (≥ 30 ng/ml)	28	22	
Vitamin D levels improved	22	20	0.76
Vitamin D levels remained same	18	22	
Vitamin D worsened	4	5	
Any Flare	8	6	0.37
Major flare	4	1	0.17
Minor flare	4	5	0.51
SLEDAI 2KG 6 months Median (IQR)	2 (4)	2 (2)	0.76
Δ SLEDAI 2KG median (min, max)	0 (− 4, 9)	0 (–4, 5)	0.81
C3 (mg/dl) median (IQR)	100.3 (35)	104 (32)	0.32
ΔC3 (mg/dl) median (IQR)	– 5.5 (24.9)	+ 2 (52)	0.59
C4 (mg/dl) median (IQR)	17.3 (11)	17.7 (10)	0.98
Δ C4 (mg/dl) median (IQR)	– 2.4 (7.9)	– 0.7 (6.6)	0.59
Low complements (C3/C4)	12	14	0.82
Improved C3/C4	6	4	0.75
Worsened C3/C4	6	7	
Anti-dsDNA median (IQR)	14.11 (51)	48.2 (135)	0.08
Δ Anti-dsDNA median (IQR)	– 15.2 (50.02)	– 2.8 (72.4)	0.06
Positive anti-dsDNA	7	16	0.87
Normalised anti-dsDNA (baseline positive- 6 months negative)	19	11	0.14
Worsening anti-dsDNA (negative at baseline and positive at 6 months)	2	4	

*C3, C4* complement component 3, 4, *SLEDAI2K* systemic lupus erythematosus disease activity index 2000, *SLEDAI 2KG* systemic lupus erythematosus disease activity index 2000 with glucocorticoids

## Discussion

In this study on a reasonably large number of patients with SLE, we found that plasma vitamin D level was insufficient in over 41% of individuals. The vitamin D levels depended on the patients’ geographical location and had no relationship to disease activity, autoantibody status or IFN-related proteins. The two study centres Lucknow (26.8467° N, 80.9462° E) and Pondicherry (11.9416° N, 79.8083° E) are located along similar longitude but differ significantly in their distance from the equator (latitudes). The patients recruited at the southern centre had higher vitamin D levels, and the proportion of those who were vitamin D deficient was also substantially lower. This trend was similar in both juvenile and adult patients.

A systematic review [23] and meta-analysis [24] have identified that patients with lupus have lower plasma vitamin D levels and a higher incidence of vitamin D deficiency compared to healthy controls. Data from India [25] and other southeast Asian countries [26] have shown extremely low vitamin D levels in the healthy population. The mean weighted vitamin D levels reported among the healthy people in India was 19.34 ± 12.08 ng/mL. A random effect meta-analysis showed that the weighted pooled prevalence of vitamin D deficiency was 67% [95% CI: 61–73%] in the community [26]. These figures are similar to the values reported in the present study. It is possible that the metadata [23, 24] on SLE overestimated the vitamin D deficiency in lupus, as the majority of the studies recruited hospital-based healthy controls. The hospital-based recruitment could have led to selection bias, and the healthy controls may not represent the general population. On the other hand, the two systematic reviews on vitamin D deficiency in the healthy population [25, 26] were mainly a synthesis of community-based studies which are likely to have systematically sampled subjects avoiding selection bias.

Even though we have not compared the vitamin D levels of our patients with those of healthy controls, the available data points to the possibility that patients with lupus may have a similar burden of vitamin D deficiency compared to their matched healthy counterparts in the community. Further, the systematic review from India [25] has also clearly identified that people from the southern part of India had higher vitamin D levels, similar to the present study findings. However, since all the patients in our study were on daily calcium supplements, which has 250 IU of vitamin D3, there is a possibility that the deficiency in our cohort was partially corrected.

The ultraviolet B (UV-B) radiation from sunlight with a wavelength between 290 and 315 nm converts 7-dehydrocholesterol to pre-vitamin D3 in the skin. This pre-vitamin D 3 is rapidly converted to cholecalciferol (vitamin D3) [27]. Less than 1% of the UV-B from the sun reaches the earth. The availability of 1% of the UV-B radiation entering the earth’s atmosphere is further determined by the distance the rays must travel to reach the earth’s surface. During winters and in areas that are away from the equator, the distance travelled by UV rays is comparatively longer. This reduces UV-B rays’ availability during winters and as one moves away from the equator [28]. The same principle is observed in our study. Patients in both the centres were recruited at equal proportions in summer and winter thus, the factor determining vitamin D levels primarily seems to be the place of residence.

Another factor that positively correlated with higher vitamin D levels was the age of the patient. It is well known that elderly individuals have lower vitamin D levels [29]. With increasing age, there is a decrease in the concentration of 7-dehydrocholesterol and a reduced response to UV radiation on the skin. Both these factors result in a lower vitamin D level in elderly individuals. In contrast to this theory, we found a positive correlation between vitamin D levels with age. This positive correlation in our study could be because > 90% of our patients were between 25 and 40 years old, an age group where bone mineral density (BMD) and vitamin D levels peak in adults [30, 31]. Moreover, the *β* coefficient was very small (0.181) for age, suggesting a negligible effect of this variable in reality.

The association studies of vitamin D levels with disease activity in lupus and interferons are variable and have produced contradicting results. While some of the studies have found an association of low vitamin D with specific clinical phenotypes, higher disease activity [6, 7] and cardiovascular co-morbidity [31] and metabolic syndrome [9] in SLE, a few others have failed to establish such associations [8, 10, 32]. The contrast may be due to the result of varying geographical, genetic and treatment-related factors [33]. Or it could be due to the small sample size that was collected over different seasons and not accounting for confounders like BMI, socio-economic status, ethnicity and sex, all of which can influence both SLE disease activity [34, 35] and vitamin D levels [36].

Increased interferon (IFN) expression in SLE modestly reflects disease activity measured by SLEDAI. Murine models of lupus have demonstrated association of low vitamin D levels with type-1 IFN gene expression and endothelial dysfunction [37]. Several human studies have in univariate analyses shown significant negative correlation of SLEDAI with IFN-α and IFN signatures [6, 38, 39]. Galectin-9 and CXCL-10 are IFN-related proteins which have proven to closely reflect IFN-related gene expression in SLE [40]. In our study, we found both Galectin-9 and CXCL-10 levels to correlate significantly with SLEDAI. This finding is similar to most other reported studies [41, 42], reemphasising the vital role of IFN in the pathogenesis of lupus. Few studies have shown significant inverse correlation of IFN alpha levels and IFN gene signature with vitamin D levels in SLE [6, 38]. However, we found no association of galectin-9 and CXCL-10 with plasma vitamin D levels, possibly because of a larger sample size and accounting for various confounding variables. Others have also reported similar non association of IFN alpha with vitamin D levels in SLE [43].

In our prospective RCT which included 91 patients followed up to 6 months, the group who received high dose vitamin D had a higher chance of achieving sufficiency in vitamin D levels than those receiving routine dose. However, no difference was observed in terms of improvement in disease activity or prevention of lupus flare. A recently published study showed improvement in disease activity (SLEDAI-2K) and fatigue at 12 months of cholecalciferol supplementation [14]. Another study on juvenile SLE patients reported improvement in vitamin D levels and bone microarchitecture among those who received cholecalciferol for 24 weeks [44]. There has been no study showing decrease in IFN gene expression with vitamin D supplementation despite achieving adequate serum levels [45]. All these studies, including the current study, emphasise that vitamin D levels can normalise with oral supplementation and is safe. However, the effect on lupus activity and IFN signature are still not established. This could be because of the variable duration of several studies and the use of background immunosuppressive agents whose effect might be so high that added impact of vitamin D may not be evident.

The strength of our study is the large sample size with details about the usual confounders for vitamin D levels. Moreover, the samples were collected at the beginning of recruitment into the cohort, thus decreasing the effect of background immunosuppressive agents. The sample collection in both the centres was conducted round the year, which negates the effect of seasonal variation in vitamin D levels. However, the effect of drugs administered before their entry into the cohort and the patients’ dietary habits are not accounted for. Still, as the patients had a short duration of symptoms, this factor may not have influenced our results. Another drawback is we have not collected data on vascular health and atherosclerotic disease. This association seems to be strong from recently published studies. Another drawback was the premature termination of the trial due to the COVID pandemic. Larger sample size with a longer duration of follow-up would be required to obtain meaningful data on the effect of vitamin D supplementation on disease activity. With the current study’s data, it would be important to determine the effect of ethnicity, vitamin D receptor polymorphisms and alternate methods of vitamin D administration in lupus.

## Conclusion

Vitamin D deficiency is common in SLE, and the prevalence may be similar to that encountered in the community. The geographical location of residence is the primary determinant of plasma vitamin D levels rather than the disease activity. The IFN-regulated proteins galectin-9 and CXCL-10 reflect disease activity independent of vitamin D levels. High-dose oral vitamin D supplementation seems safe and more effective in improving vitamin D levels in SLE, but its role in modifying disease progress will need further studies.

## Supplementary Information

**Additional file 1: Supplementary Table 1.** Baseline variables of the cohort.

**Additional file 2: Supplementary Table 2.** Multiple linear regression model for predicting vitamin D levels in the cohort.

**Additional file 3: Supplementary Table 3.** Baseline parameters of randomised patients.

## Data Availability

All the data will be made available upon reasonable request to the corresponding author.
