# Supplementary material for: Clinical and serological association of plasma 25-hydroxyvitamin D (25(OH)D) levels in lupus and the short-term effects of oral vitamin D supplementation
Source: Arthritis Res Ther. 2023 Jan 3;25:2. doi: 10.1186/s13075-022-02976-7 (PMC9807987; doi:10.1186/s13075-022-02976-7)
Supplement: Supplementary file 1 — Additional file 1: Supplementary Table 1. Baseline variables of the cohort. [file 13075_2022_2976_MOESM1_ESM.docx]

| Supplementary Table 1: Baseline variables of the cohort | |
| --- | --- |
| Parameter | N=702 |
| Age in years  (Mean ±SD) | 29.46 ±10.7 |
| Females, n (%) | 653 (93%) |
| North India, n(%) | 304 (43.4%) |
| South India, n(%) | 398 (56.6%) |
| Juvenile , n(%) | 103 (14.3%) |
| Duration of SLE in months  median (IQR) | 16 (28) |
| BMI (Mean±SD) | 21.49 ± 4.95 |
| Plasma Vitamin-D,  median (IQR) ng/ml | 22.83 (28) |
| Vitamin D Deficient ( ≤20 ng/ml), n(%) | 291 (41.5%) |
| Vitamin D Insufficient ( 20.1-29.99) ng/ml), n(%) | 208 (29.5%) |
| Vitamin D Sufficient ( ≥30 ng/ml) , n(%) | 203 (29%) |
| SLEDAI 2K at baseline, median (IQR) | 10 (12) |
| SLEDAI 2K> 4, n(%) | 484 (69%) |
| C3 mg/dl  Median (IQR) | 65.7 (58.75) |
| Low C3, n(%) | 447 (63.8%) |
| C4 mg/dl  Median (IQR) | 11.5 (12.67%) |
| Low C4, n(%) | 317 (45.2%) |
| Low complements (C3/ C4), n(%) | 490 (69.9) |
| Anti dsDNA, (Median, IQR) IU/mL | 215.1 (614.3) |
| Positive anti-dsDNA, n(%) | 467 (66.6%) |
| CXCL-10 pg/ml, median(IQR) (n=474) | 141.58 (248.08) |
| Galectin 9 ng/ml, median(IQR) (n-548) | 11.940 (17.44) |
| Constitutional, n(%) | 544 (77.6%) |
| Renal, n(%) | 283 (40.4%) |
| CNS, n(%) | 113 (16.1%) |
| Mucocutaneous, n(%) | 634 (90.4%) |
| BMI- body mass index, chemokine (C-X-C motif) ligand, CNS- central nervous system, dsDNA- Double stranded Deoxyribose Nucleic acid, SLEDAI-Systemic lupus erythematosus disease activity index, * p<0.05 | |
