# Supplementary material for: Clinical and serological association of plasma 25-hydroxyvitamin D (25(OH)D) levels in lupus and the short-term effects of oral vitamin D supplementation
Source: Arthritis Res Ther. 2023 Jan 3;25:2. doi: 10.1186/s13075-022-02976-7 (PMC9807987; doi:10.1186/s13075-022-02976-7)
Supplement: Supplementary file 2 — Additional file 2: Supplementary Table 2. Multiple linear regression model for predicting vitamin D levels in the cohort. [file 13075_2022_2976_MOESM2_ESM.docx]

| Supplementary Table 2: Multiple linear regression model for predicting vitamin D levels in the cohort | | | | | | | |
| --- | --- | --- | --- | --- | --- | --- | --- |
| Model | Unstandardised Coefficients | | Standardised Coefficients | t | Sig. | 95.0% Confidence Interval for B | |
|  | Beta | Std. Error | Beta |  |  | Lower Bound | Upper Bound |
| (Constant) | 13.176 | 3.657 |  | 3.603 | .000 | 5.996 | 20.356 |
| SLEDAI 2K | -.086 | .084 | -.047 | -1.034 | .302 | -.251 | .078 |
| Age | .181 | .058 | .130 | 3.135 | .002* | .068 | .294 |
| DOD (M) | .002 | .014 | .006 | .134 | .894 | -.026 | .030 |
| BMI | -.018 | .127 | -.006 | -.145 | .885 | -.268 | .231 |
| Centre^#^ | 4.369 | 1.165 | .145 | 3.750 | .000* | 2.081 | 6.656 |
| C3 Value | .011 | .019 | .028 | .573 | .567 | -.026 | .048 |
| C4 value | .016 | .061 | .012 | .261 | .794 | -.104 | .136 |
| Dependent Variable: Vitamin D, BMI- body mass index, C3, C4- Complement component 3, 4, DOD- Duration of disease, SLEDAI2K- Systemic lupus erythematosus disease activity index 2000. * p<0.05  # Centre refers to the site of patient recruitment | | | | | | | |
