# Supplementary material for: Clinical and serological association of plasma 25-hydroxyvitamin D (25(OH)D) levels in lupus and the short-term effects of oral vitamin D supplementation
Source: Arthritis Res Ther. 2023 Jan 3;25:2. doi: 10.1186/s13075-022-02976-7 (PMC9807987; doi:10.1186/s13075-022-02976-7)
Supplement: Supplementary file 3 — Additional file 3: Supplementary Table 3. Baseline parameters of randomised patients. [file 13075_2022_2976_MOESM3_ESM.docx]

| **Supplementary Table 3: Baseline parameters of randomised patients** | | | |
| --- | --- | --- | --- |
| **Parameter** | **High dose (n=87)** | **Routine dose (n=85)** | **P value** |
| Age in years  (Mean ±SD) | 34.83(9.9) | 34.42 (9.2) | 0.78 |
| Sex (Females) | 83 | 78 | 0.36 |
| Duration of SLE in years  median (IQR) | 6 (7) | 5 (6.75) | 0.36 |
| Sunlight exposure in hours/day  median (IQR) | 0.5 (0.5) | 0.88 (1.5) | 0.45 |
| BMI (Mean ± SD) | 24.03 (4.7) | 23.79 (6.2) | 0.77 |
| Vit D at Baseline  median (IQR) ng/ml | 25.63 (12) | 26.47 (15) | 0.68 |
| Vitamin D Deficient ( ≤20 ng/ml) | 8 | 7 | 0.67 |
| Vitamin D Insufficient ( 21-29 ng/ml) | 43 | 41 |  |
| Vitamin D Sufficient ( ≥30 ng/ml) | 19 | 25 |  |
| SLEDAI 2KG @baseline  median (IQR) | 2 (2) | 2 (3.5) | 0.66 |
| SLICC ACR at baseline  median (IQR) | 0.06 (0.34)  0 (0-2) | 0.01 (0.12)  0 (0-1) | 0.98 |
| C3 mg/dl  Median (IQR) | 106 (35.1) | 102 (31.7) | 0.82 |
| Low C3 | 12 | 13 | 1.0 |
| C4 mg/dl  Median (IQR) | 21.5 (12) | 18.7 (16) | 1.0 |
| Low C4 | 18 | 19 | 1.0 |
| Low complements (C3/ C4) | 26 | 25 | 0.87 |
| anti-dsDNA  median (IQR) | 25.9 (60.2) | 37.4 (107.1) | 0.7 |
| Positive anti-dsDNA | 31 | 27 | 0.62 |
| Renal | 39 | 44 | 0.36 |
| CNS | 8 | 9 | 0.8 |
| Haematological | 45 | 37 | 0.29 |
| Mucocutaneous | 75 | 81 | 0.06 |
